# Supplementary material for: Endoplasmic reticulum stress promotes sorafenib resistance via miR-188-5p/hnRNPA2B1-mediated upregulation of PKM2 in hepatocellular carcinoma
Source: Mol Ther Nucleic Acids. 2021 Oct 1;26:1051–65. doi: 10.1016/j.omtn.2021.09.014 (PMC8569435; doi:10.1016/j.omtn.2021.09.014)
Supplement: Document S1. Tables S1–S4 [file mmc1.pdf]

OMTN, Volume *26*

Supplemental information

Endoplasmic reticulum stress promotes sorafenib resistance via miR-188-5p/hnRNPA2B1-mediated upregulation of PKM2 in hepatocellular carcinoma

Bei Zhou, Donghui Lu, Anqi Wang, Jie Cui, Li Zhang, Jian Li, Lulu Fan, Wei Wei, Jiatao Liu, and Guoping Sun

Supplemental table 1. Correlations between PKM2 and ER-stress related proteins expression in HCC patients

ER-stress related proteins expression	PKM2 expression		R^2	*P*-value
	Low (N=34)	High (N=38)		
GRP78 expression			0.0663	0.0291*
Low(N=37)	22	15		
High(N=35)	12	23		
PERK expression			0.0874	0.0117*
Low(N=37)	21	16		
High(N=35)	13	22		
IRE1α expression			0.1291	0.0019**
Low(N=32)	21	11		
High(N=40)	13	27		
ATF6 expression			0.0764	0.0188*
Low(N=33)	21	12		
High(N=39)	13	26		

GRP78: glucose-regulated protein; PERK: PKR-like ER stress kinase; IRE1α: inositol-requiring transmembrane kinase and endonuclease 1α; ATF6: activating transcription factor 6; **P < 0.01, *P < 0.05.

Supplemental table 2. Correlations between ER-stress related proteins or PKM2 and hnRNPA2B1 expression in HCC patients

ER-stress related proteins or PKM2 expression	hnRNPA2B1 expression		$\chi 2$	*P*-value
	Low (N=34)	High (N=38)		
GRP78 expression			4.573	0.032*
Low(N=37)	22	15		
High(N=35)	12	23		
PERK expression			12.642	0.000**
Low(N=37)	25	12		
High(N=35)	9	26		
IRE1 expression			22.071	0.000**
Low(N=32)	25	7		
High(N=40)	9	31		
ATF6 expression			9.242	0.002**
Low(N=33)	22	11		
High(N=39)	12	27		
PKM2 expression			10.783	0.001**
Low(N=34)	23	11		
High(N=38)	11	27		

hnRNPA2B1: heterogeneous nuclear ribonucleoprotein A2/B1. **P < 0.01, *P < 0.05.

Supplemental table 3. Clinicopathologic features of HCC patients depending on hnRNPA2B1 expression

Clinical pathological factors	Total (N=72)	hnRNPA2B1 expression Low (N=34)	hnRNPA2B1 expression High (N=38)	χ2	*P*-value
Gender，N(%)				0.086	0.770
M	20(27.7)	10(29.4)	10(26.3)		
F	52(72.2)	24(70.6)	28(73.7)		
Age，N(%)				0.350	0.554
≤60 y	44(62.5)	22(64.7)	22(57.9)		
＞60 y	28(37.5)	12(35.3)	16(42.1)		
History of hepatitis, N(%)				0.009	0.992
N	25(34.7)	12(35.3)	13(34.2)		
Y	47(65.3)	22(64.7)	25(65.8)		
History of cirrhosis，				1.006	0.316
N	40(55.6)	21(61.8)	19(50.0)		
Y	32(44.4)	13(38.2)	19(50.0)		
Tumor size，N(%)				6.019	0.067
＜5cm	18(25.0)	13(38.2)	5(13.2)		
5-10cm	36(50.0)	14(41.2)	22(57.9)		
≥10cm	18(25.0)	7(20.6)	11(28.9)		
Clinical stages，N(%)				0.991	0.320
Ⅰ/Ⅱ	51(70.8)	26(76.5)	25(65.8)		
Ⅲ/Ⅳ	21(29.2)	8(23.5)	13(34.2)		
Degree of differentiation，N(%)				7.155	0.026*
high	20(27.8)	13(38.2)	7(18.4)		
moderate	34(47.2)	17(50.0)	17(44.7)		
poor	18(25.0)	4(11.8)	14(36.8)		
AFP value, N(%)				1.935	0.164
＜400 ng/mL	47(65.3)	25(73.5)	22(57.9)		
≥400 ng/mL	25(34.7)	9(26.5)	16(42.1)		

Supplemental table 4. Oligonucleotide Sequences

Oligonucleotide	Sequences Sense (5'-3')	Sequences Antisense (5'-3')
mimic negative control	UUCUCCGAACGUGUCACGUTT	ACGUGACACGUUCGGAGAATT
miR-188-5p mimics	CAUCCCUUGCAUGGUGGAGGG	UUGUAGGGAACGUACCACCUC
inhibitor negative control	CAGUACUUUUGUGUAGUACAA	

miR-188-5p inhibitor	CCCUCCACCAUGCAAGGGAUG	
hnRNPA2B-primer-1	CAGGGTAGTTGAGCCAAAACG	TTCCAGACTGCCTATCGGTAA
hnRNPA2B-primer-2	AAGAAATGCAGGAAGTCCAAAGT	CTCCTCCATAACCAGGGCTAC
hsa-miR-188-5p	GCGCATCCCTTGCATGGT	AGTGCAGGGTCCGAGGTATT
miR-33b-3p	GCAGTGCCTCGGCAGTG	AGTGCAGGGTCCGAGGTATT
miR-222-5p	CGCGCTCAGTAGCCAGTGTA	AGTGCAGGGTCCGAGGTATT
hsa-miR-301a-5p	CGCGGCTCTGACTTTATTGC	AGTGCAGGGTCCGAGGTATT
hsa-miR-675-5p	GGTGCGGAGAGGGCCC	AGTGCAGGGTCCGAGGTATT
hsa-miR-877-3p	CGCGTCCTCTTCTCCCTCC	AGTGCAGGGTCCGAGGTATT
hsa-miR-1247-5p	GACCCGTCCCGTTCGTC	AGTGCAGGGTCCGAGGTATT
hsa-miR-1257	CGCGAGTGAATGATGGGTT	AGTGCAGGGTCCGAGGTATT
hsa-miR-7975	CGCGCGATCCTAGTCACG	AGTGCAGGGTCCGAGGTATT
