# Supplementary material for: Endoplasmic reticulum stress promotes sorafenib resistance via miR-188-5p/hnRNPA2B1-mediated upregulation of PKM2 in hepatocellular carcinoma
Source: Mol Ther Nucleic Acids. 2021 Oct 1;26:1051–65. doi: 10.1016/j.omtn.2021.09.014 (PMC8569435; doi:10.1016/j.omtn.2021.09.014)
Supplement: Document S2. Article plus supplemental information [file mmc2.pdf]

Molecular Therapy
Nucleic Acids
Original Article

Endoplasmic reticulum stress promotes sorafenib resistance via miR-188-5p/hnRNPA2B1-mediated upregulation of PKM2 in hepatocellular carcinoma

Bei Zhou,[1,4] Donghui Lu,[1,4] Anqi Wang,[1,4] Jie Cui,[1] Li Zhang,[1] Jian Li,[1] Lulu Fan,[1] Wei Wei,[2] Jiatao Liu,[1,3] and Guoping Sun[1]

[1]Department of Oncology, the First Affiliated Hospital of Anhui Medical University, Hefei 230022, Anhui, China; [2]Institute of Clinical Pharmacology, Anhui Medical University, Hefei 230032, Anhui, China; [3]Department of Pharmacy, the First Affiliated Hospital of Anhui Medical University, Hefei 230022, Anhui, China

Emerging evidence has shown that endoplasmic reticulum (ER) stress promotes sorafenib resistance in hepatocellular carcinoma (HCC). However, the underlying mechanisms are poorly understood. The purpose of this study was to explore the mechanism by which ER stress promotes sorafenib resistance in HCC. We found that pyruvate kinase isoform M2 (PKM2) was highly expressed in human HCC tissues and co-related with worse clinicopathologic features and overall survival. Activation of ER stress positively correlated with PKM2 expression both in HCC tissue samples and tunicamycin (TM)-induced HCC cell lines. PKM2 knockdown increased sorafenib-induced apoptosis and decreased the ability of colony formation, while upregulation of PKM2 reverses this phenomenon. Furthermore, high-throughput sequencing identified that activation of ER stress significantly downregulated the expression of miR-188-5p in HCC cells. According to bioinformatics analysis and dual-luciferase assays, we further confirmed that *hnRNPA2B1* is the target gene of miR-188-5p. Downregulating the expression of hnRNPA2B1 with siRNA could decrease the expression of PKM2 and enhance sorafenib-induced apoptosis in HepG2 cells. Our study demonstrated that ER stress could promote sorafenib resistance through upregulating PKM2 via miR-188-5p/hnRNPA2B1. Therefore, targeting the miR-188-5p/hnRNPA2B1/PKM2 pathway and ER stress may prove instrumental in overcoming sorafenib resistance in HCC treatment.

INTRODUCTION

Hepatocellular carcinoma (HCC) is one of the most malignant tumors that seriously endanger human health. The overall survival of advanced HCC patients is extremely short, and the 5-year survival rate is <10%. In recent years, the incidence and mortality of HCC has been increasing as is evident by the >800,000 new cases and >750,000 patients died of this disease worldwide in 2019.[1] In the past few decades, there has been no encouraging breakthrough in the treatment of HCC, mainly because liver cancer cells generate apoptosis resistance to a variety of therapeutic drugs.[2] Sorafenib, a multi-target molecular drug, is one of the main strategies for the treatment of advanced HCC, which can extend a patient's overall survival by inhibiting the rapid proliferation of tumor cells and the formation of tumor blood vessels. The mechanism of sorafenib primarily associates with the suppression of various receptor tyrosine kinases and blockage of the VEGF/Raf/MER/ERK-mediated multiple signal pathways.[3] However, >70% of patients with advanced HCC did not benefit from the initial treatment of sorafenib, probably because of primary resistance; simultaneously, most patients with effective initial treatment acquired sorafenib resistance within 1 year.[4] Therefore, exploring the mechanisms of sorafenib resistance in the treatment of HCC has valuable clinical significance.

Endoplasmic reticulum (ER) stress is characterized by the abnormal aggregation of plentiful unfolded and misfolded proteins in the lumen of the ER due to imbalances in intracellular energy levels, redox state, and calcium ion concentration.[5] To alleviate ER stress, the unfolded protein response (UPR) pathways, including PERK, ATF6, and IRE1α were subsequently activated.[6] Moderate ER stress is conducive to tumor cell survival and anti-apoptosis.[7] Our previous research showed that temperate ER stress reduced the sensitivity of HCC to sorafenib.[8] ER stress relieves the organelle and protein damage induced by sorafenib to mediate apoptosis resistance; however, the specific mechanism of sorafenib resistance mediated by ER stress in HCC is unknown. Our research direction is to explore whether ER stress can affect energy metabolism reprogramming to increase intracellular energy production and then mediate sorafenib resistance.

Different from normal cells, most tumor cells tend to obtain energy from a higher glycolysis rate, even in the condition of sufficient oxygen supply. This kind of energy metabolism reprogramming is called

Received 18 November 2020; accepted 24 September 2021;
https://doi.org/10.1016/j.omtn.2021.09.014.

[4]These authors contributed equally

Correspondence: Jiatao Liu, PhD, Departments of Oncology and Pharmacy, the First Affiliated Hospital of Anhui Medical University, Hefei 230022, Anhui, China.
E-mail: liujiatao@ahmu.edu.cn
Correspondence: Guoping Sun, PhD, Department of Oncology, the First Affiliated Hospital of Anhui Medical University, Hefei 230022, Anhui, China.
E-mail: sungp@ahmu.edu.cn

This is an open access article under the CC BY license (http://creativecommons.org/licenses/by/4.0/).

the Warburg effect.[9] Pyruvate kinase isoform M2 (PKM2) is an essential enzyme that mediates glycolysis and is mainly expressed in tumor cells and embryonic cells. Another translation product of *PKM* gene, PKM1, is elementarily expressed in normally differentiated tissues.[10] Méndez-Lucas et al.[11] showed that PKM2 mediated the reprogramming of glucose metabolism, enabling tumor cells to obtain energy quickly and directly, which is beneficial for tumor cells to acquire advantageous survival conditions and apoptosis resistance. PKM2 participates in the resistance of different tumor cells to chemotherapy drugs, including sorafenib.[12] Both ER stress and PKM2 are involved in the pro-survival mechanism and apoptosis resistance of tumor cells; therefore, the relationship between them aroused our great research interest.

Studies have shown that ER stress modulates the microRNA (miRNA) expression profile of tumor cells to regulate their biological behavior.[13] Our previous studies have shown that ER stress can regulate programmed cell death protein-ligand 1 (PD-L1) expression of immune cells by upregulating miR-23a-3p, which was contained in exosomes of liver cancer cells.[14] miRNAs are a class of non-coding small RNAs with $\sim$22 nucleotides in eukaryotes. They are usually involved in post-transcriptional regulation and mRNA translation inhibition of target genes by directing the target mRNA to break at specific sites or recognizing and binding to the 3′ UTR of the mRNA of the target gene.[15] miRNA plays an indispensable role in the regulation of various biological processes of tumor cells, including cell proliferation and apoptosis, energy metabolism reprogramming, and tumor invasion and metastasis.[16,17] Studies have shown that miRNA enhances the resistance of tumor cells to chemotherapy drugs by targeting key proteins in the biological behavior of tumor cells.[18] Our research focuses on whether ER stress regulates the biological behaviors of tumor cells, including energy metabolism and apoptosis resistance by modifying the miRNA expression profile of liver cancer cells.

The expression of PKM2 in HCC and its role in apoptosis resistance remains unclear. Research on the connections between the key metabolic enzyme PKM2 in the process of aerobic glycolysis and ER stress are exceedingly limited. In this study, experimental evidence revealed that ER stress upregulates PKM2 expression via miR-188-5p/hnRNPA2B1 to promote sorafenib resistance in HCC. It also indicates that PKM2, when regulated by ER stress, may be a potential therapeutic target to overcome sorafenib resistance that has emerged in HCC.

RESULTS

Overexpression of PKM2 correlates with worse clinicopathologic features in HCC patients

PKM2 has been shown to overexpress in several types of tumors and be involved in the malignant biological behavior of tumor cells, including proliferation, apoptosis resistance, invasion, and migration.[19,20] To understand the expression and role of PKM2 in HCC, we collected freshly removed HCC tissues and adjacent tissues from four liver cancer patients, and western blot analysis showed that the expression of PKM2 in cancerous tissues was significantly increased compared with adjacent normal liver tissues (Figure 1A). Meanwhile, immunohistochemical analysis of HCC tissues and paired liver tissues from 72 patients found that PKM2 was mainly expressed in the cytoplasm with brown staining, and the expression of PKM2 was significantly upregulated in HCC tissues compared with corresponding normal liver tissues (Figure 1B). PKM2 expression levels were analyzed semiquantitatively by scoring the immunohistochemistry (IHC) results according to staining intensity and the percentage of positively stained cells. We found that the IHC scores in HCC tissues were evidently higher than that in the adjacent tissues (IHC scores ≥ 10 mean PKM2 high expression, 38/72 versus 0/72) (Figure 1C). Furthermore, the relationship between the protein level of PKM2 and the clinicopathological data of the 72 HCC patients is presented in Table 1. We found that HCC patients with a higher expression of PKM2 demonstrated a lower degree of differentiation (poor and moderate differentiation, 86.8% versus 55.9%, $p = 0.015$) and a larger volume (tumor size ≥ 5 cm, 89.1% versus 58.8%, $p = 0.010$) of tumor tissue. As shown in Figure 1D, we also found that HCC patients with a higher expression of PKM2 had a shorter overall survival (median survival, 29.5 versus 16.5 months, $p = 0.0351$). We also detected the expression of PKM2 and PKM1 in several liver cancer cell lines, including HepG2, Hep3B, Huh7, and SMMC-7721, and liver cell line L02. Western blot analysis found that the protein level of PKM2 in liver cancer cell lines was visibly higher compared with L02 cells, while PKM1 was mainly expressed in normal liver cells. Among these liver cancer cell lines, SMMC-7721 had the highest expression of PKM2, followed by Huh7 and HepG2, and Hep3B had the lowest expression of PKM2 (Figures 1E and 1F). This was also verified in immunofluorescence analysis. Fluorescence intensity semiquantitative analysis indicated that the expression of PKM2 in HepG2 cells cytoplasm was significantly higher than that of PKM1. However, L02 cells express high levels of PKM1 instead of PKM2 (Figures 1G and 1H).

Upregulation of PKM2 contributes to sorafenib resistance in HCC cells

PKM2 was reported to play an important role in chemotherapy resistance in some tumor cells.[21] To assess the role of PKM2 in sorafenib resistance in the process of HCC treatment, small interfering RNA (siRNA) and overexpressing plasmid transfection were used to, respectively, silence and overexpress PKM2 in different HCC cell lines. We observed the proliferation of different HCC cell lines in different concentrations of sorafenib. The MTT assay results indicated that the IC_{50} (50% inhibiting concentration) of SMMC-7721, Huh7, HepG2, and Hep3B were 17.465, 14.589, 11.968, and 9.395 μM, respectively. We found that the higher the expression of PKM2 of HCC cells, the weaker the inhibitory effect of sorafenib on the proliferation of HCC cells (Figure 2A). We further confirmed the efficiency of PKM2 knockdown by three siRNA in Huh7 cells and selected the most effective interfering RNA (si-PKM2–3) for the following functional experiment (Figures 2B and 2C). The flow cytometry results showed that cells stained with annexin-V-fluorescein isothiocyanate (FITC) and/or phosphatidylinositol (PI) were

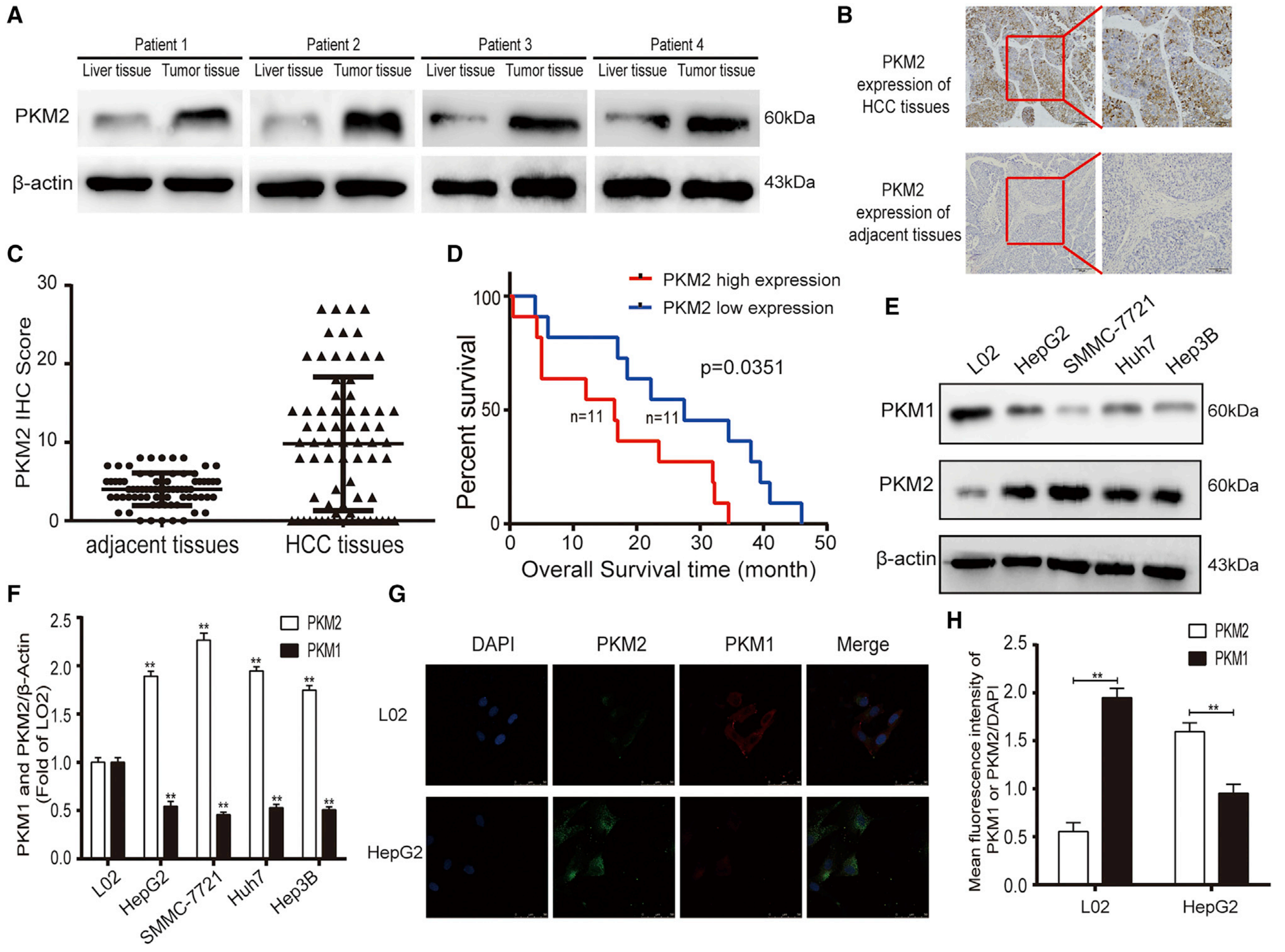

Figure 1. Overexpression of PKM2 correlates with worse clinicopathologic features in HCC patients

(A) The protein level of PKM2 in human liver tissues and HCC tissues was detected by western blot. (B and C) Representative expression and immunohistochemistry score of PKM2 in HCC and adjacent tissue specimens (100× and 200× magnification). (D) The Kaplan-Meier method was applied in overall survival analysis between the PKM2 high-expression and PKM2 low-expression groups. (E and F) The expression levels of PKM2 and PKM1 in different HCC cells were analyzed by western blot. (G and H) Immunofluorescence analysis of the location and fluorescence intensity semiquantitative analysis of the expression of PKM2 and PKM1 in HepG2 and L02 cells (40× magnification). $^{**}p < 0.01$ (n = 3).

apoptotic cells. The inhibited PKM2 expression induced by siRNA could increase sorafenib-induced apoptosis in Huh7 cells, which have a higher expression of PKM2 (Figures 2D and 2E). Furthermore, knocking down PKM2 in the presence of sorafenib decreased the ability of Huh7 cells to form colonies (Figures 2F and 2G). In contrast, to determine whether PKM2 overexpression enhances the sensitivity of liver cancer cells to sorafenib, the overexpressing plasmids GV230-PKM2 were transfected into HepG2 cells, which have a lower expression of PKM2. Western blot assay was used to confirm the effect of GV230-PKM2 in the overexpression of PKM2 (Figures 2H and 2I). Two-channel flow cytometry apoptosis assays show that GV230-PKM2 decreased the percentage of early- and advanced-stage apoptotic cells in the treatment of sorafenib (Figures 2J and 2K). Meanwhile, PKM2 overexpression increased the ability of HepG2 cells to form colonies in the presence of sorafenib (Figures 2L and 2M). These results suggested that PKM2 may play an indispensable role in sorafenib resistance in the treatment of HCC.

Overexpression of PKM2 is closely related to activation of ER stress in HCC

Our previous research showed that ER stress mediated sorafenib resistance in HCC.[8] To test whether ER stress promotes sorafenib resistance by regulating the activation of PKM2, we explored whether there was a connection between overexpressed PKM2 and activated ER stress in HCC. Immunohistochemical analysis of 72 HCC specimens was used to evaluate the protein levels of PKM2 and ER stress-related proteins, including IRE1α, ATF6, PERK, and GRP78 (Figures 3A and 3B). In comparison with HCC samples with a low level of PKM2, we observed that GRP78 was highly expressed in HCC tissues with PKM2 high expression (12/34 versus 23/38, p = 0.0291), as well as ATF6

Table 1. Clinicopathologic features of HCC patients, depending on PKM2 expression

Clinical pathological factors	Total (N = 72)	PKM2 expression		χ^2	p
		Low (N = 34)	High (N = 38)		
Gender, N (%)				0.580	0.446
Male	52 (72.2)	26 (76.5)	26 (68.4)		
Female	20 (27.8)	8 (23.5)	12 (31.6)		
Age, y, N (%)				0.350	0.554
≤60 y	44 (62.5)	22 (64.7)	22 (57.9)		
>60 y	28 (37.5)	12 (35.3)	16 (42.1)		
History of hepatitis, N (%)				1.935	0.164
N	25 (34.7)	9 (26.5)	16 (42.1)		
Y	47 (65.3)	25 (73.5)	22 (57.9)		
History of cirrhosis, N (%)				1.884	0.170
N	40 (55.6)	16 (47.1)	24 (63.2)		
Y	32 (44.4)	18 (52.9)	14 (36.8)		
Tumor size, cm, N (%)				9.139	0.010*
<5	18 (25.0)	14 (41.2)	4 (10.5)		
5–10	36 (50.0)	14 (41.2)	22 (57.9)		
≥10	18 (25.0)	6 (17.6)	12 (31.6)		
Clinical stages, N (%)				0.991	0.320
I/II	51 (70.8)	26 (76.5)	25 (65.8)		
III/IV	21 (29.2)	8 (23.5)	13 (34.2)		
Degree of differentiation, N (%)				8.634	0.015*
High	20 (27.7)	15 (44.1)	5 (13.2)		
Moderate	34 (47.3)	12 (35.3)	22 (57.9)		
Poor	18 (25.0)	7 (20.6)	11 (28.9)		
AFP value, ng/Ml, N (%)				0.160	0.690
<400	47 (65.3)	23 (67.6)	24 (63.2)		
≥400	25 (34.7)	11 (32.4)	14 (36.8)		

AFP, α-fetoprotein; HCC, hepatocellular carcinoma; PKM2, pyruvate kinase isoform M2.
*$p < 0.05$.

(13/34 versus 26/38, p = 0.0188), PERK (13/34 versus 22/38, p = 0.0117), and IRE1 (13/34 versus 27/38, p = 0.0019) (Figure 3C; Table S1). Similarly, immunofluorescence double-staining analysis visually showed that PKM2 (green) and GRP78 (red) were highly co-expressed in HepG2 cells (Figure 3D). Overall, these results revealed there exists a close relationship between PKM2 expression and ER stress.

Activated ER stress in HCC triggers the expression of PKM2

Since PKM2 expression is closely related to ER stress-related protein expression, we further investigated the precise crosstalk between PKM2 and ER stress. First, an ER stress inducer, tunicamycin (TM), was applied to observe the effect of activated ER stress on PKM2 expression. Western blot results showed that the expression of PKM2 was increased obviously in HepG2 cells treated with different concentrations of TM for various times. However, PKM1 was barely expressed in HepG2 cells (Figures 4A and 4B). Consistently, immunofluorescence analysis showed that TM increased the expression of PKM2 based on enhanced green fluorescence intensity in the cytoplasm of HepG2 cells (Figure 4C). Second, ER stress was reported to regulate downstream genes through the UPR pathway to affect the biological behavior of tumor cells.[22] We further investigated whether ER stress regulates the expression of PKM2 through any of the three UPR pathways. Three UPR pathways were interfered with by transfecting with corresponding siRNA in the HepG2 cell (Figures 4D and 4E). Western blot results indicated that the expression of PKM2 did not change statistically after the three UPR pathways were interfered with by the most effective siRNA (Figures 4F and 4G). Third, to test whether PKM2 had an effect on ER stress, we regulated the expression of PKM2 to observe the changes in ER stress-related proteins. Western blot results showed that the protein levels of ER stress markers, including GRP78, IRE1α, ATF6, and PERK, were decreased after knocking down the expression of PKM2 in Huh7 cell through siRNA (Figures 4H and 4I). The above results suggest that the expression of PKM2 triggered by activated ER stress is independent of the three UPR pathways. Furthermore, PKM2 positive feedback regulates ER stress.

Activation of ER stress regulates the expression of PKM2 through miR-188-5p in HCC

ER stress has been reported to regulate important biological processes by inducing miRNA expression to promote cell survival.[23] Thus, we further investigated whether ER stress can selectively modulate the expression of the *PKM* gene to PKM2 rather than PKM1 by altering the miRNA expression profile of liver cancer cells. High-throughput sequencing analyses of ER-stressed HepG2 cell induced by TM were performed, and we found that compared with unstressed HepG2 cells, 17 miRNAs were differentially expressed in ER-stressed liver cancer cells. Among these miRNAs, the expression of 9 miRNAs (miR33b-3p, miR-188-5p, miR222-5p, miR301a-5p, miR675-5p, miR877-3p, miR1247-5p, miR1257, and miR7975) was downregulated and the expression of 8 miRNAs (miR7-1-3p, miR22-5p, miR26-2-3p, miR148a-3p, miR148a-5p, miR215-5p, miR424-3p, and miR616-5p) was upregulated in ER-stressed liver cancer cells (Figure 5A). Recent studies and bioinformatics analysis have manifested that 47 miRNAs could regulate the energy metabolism reprogramming of tumor cells, 4 of them (miR33b-3p, miR-188-5p, miR22-5p, and miR301a-5p) expressed differentially in ER-stressed HCC cells.[24–26] Furthermore, since PKM2 is upregulated in HCC, qRT-PCR (quantitative real-time-PCR) was used to verify the expression of nine downregulated miRNAs. The result revealed that 5 of the downregulated miRNAs in the ER-stressed cells had statistical differences (miR-188-5p, miR222-5p, miR877-3p, miR1257, and miR33b-3p) and miR-188-5p had the highest fold induction (0.375-fold) in ER-stressed HepG2 cells compared with unstressed HepG2 cells (Figures 5B and 5C). Therefore, we investigated whether miR-188-5p could regulate the expression of PKM2. Western blot results showed that the transfection of miR-188-5p mimic downregulated the expression of PKM2 in HepG2 cell (Figures 5D and 5E). Simultaneously, we further investigated the effect

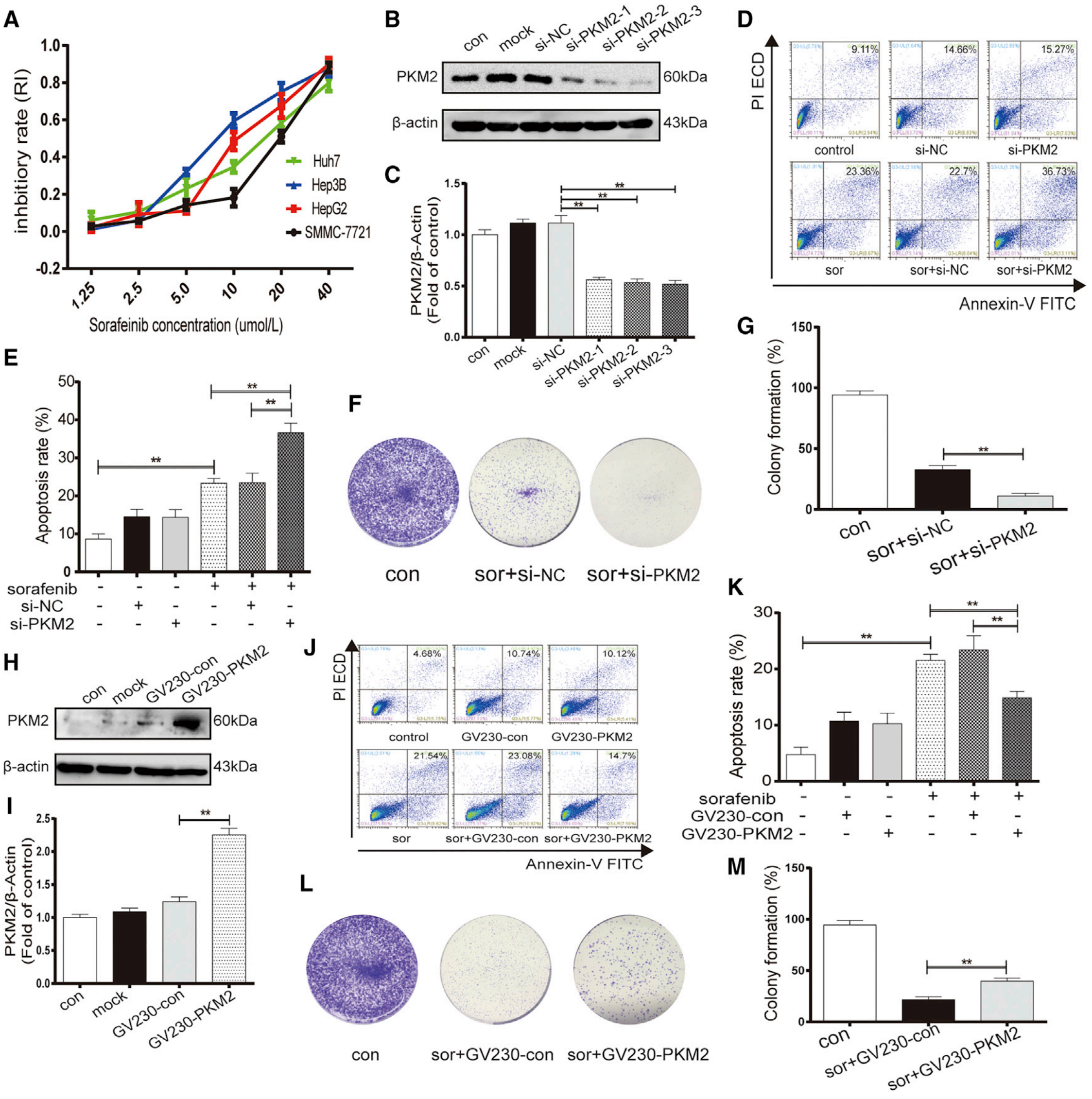

Figure 2. Upregulation of PKM2 contributes to sorafenib resistance in HCC cells

(A) MTT assay analysis of cell viability of different HCC cells when exposed to sorafenib (1.25, 2.5, 5.0, 10, 20, and 40 μM) for 24 h. (B, C, H, and I) Western blot analysis of the efficiency of PKM2 knockdown (B and C) and overexpression (H and I). (D, E, J, and K) Flow cytometry analysis of apoptosis rate in Huh7 cells treated with sorafenib (10 μM, 24 h) after transfecting with PKM2 siRNA or si-NC (siRNA-negative control) for 24 h (D and E) and in HepG2 cells treated with sorafenib (10 μM, 24 h) after transfecting with overexpressed plasmid GV230-PKM2 or GV230-control for 24 h (J and K). (F, G, L, and M) Colony formation assay analysis of cell ability in Huh7 cells and HepG2 cells. **$p < 0.01$ (n = 3).

of miR-188-5p in sorafenib resistance of liver cancer cells. Flow cytometry results demonstrated that apoptosis induced by sorafenib significantly upregulated after HepG2 cell transfected with miR-188-5p mimic (Figures 5F and 5G). These results indicate that ER stress in HCC regulates the expression of PKM2 through miR-188-5p, which mediates sorafenib resistance in liver cancer cells.

hnRNPA2B1 is a direct target of miR-188-5p

On the basis of the database that predicts and validates miRNA targets, including TargetScan (www.targetscan.org), miRDB database (http://mirdb.org), and miRWalk (http://mirwalk.umm.uni-heidelberg.de/), *PKM* is not the direct target gene of miR-188-5p. We believe that there exists a direct target gene of miR-188-5p that acts as a bridge between miR-188-5p and PKM2. Among the predicted target genes of miR-188-5p, the heterogeneous nuclear ribonucleoprotein (hnRNP) family, including hnRNPA0, hnRNPA1, and hnRNPA2B1, are involved in the splicing of *PKM* genes, and hnRNPA2B1 was predicted by multiple databases.[27] Bioinformatics analysis found that the hnRNPA2B1 mRNAs contained a complementary 3′ UTR

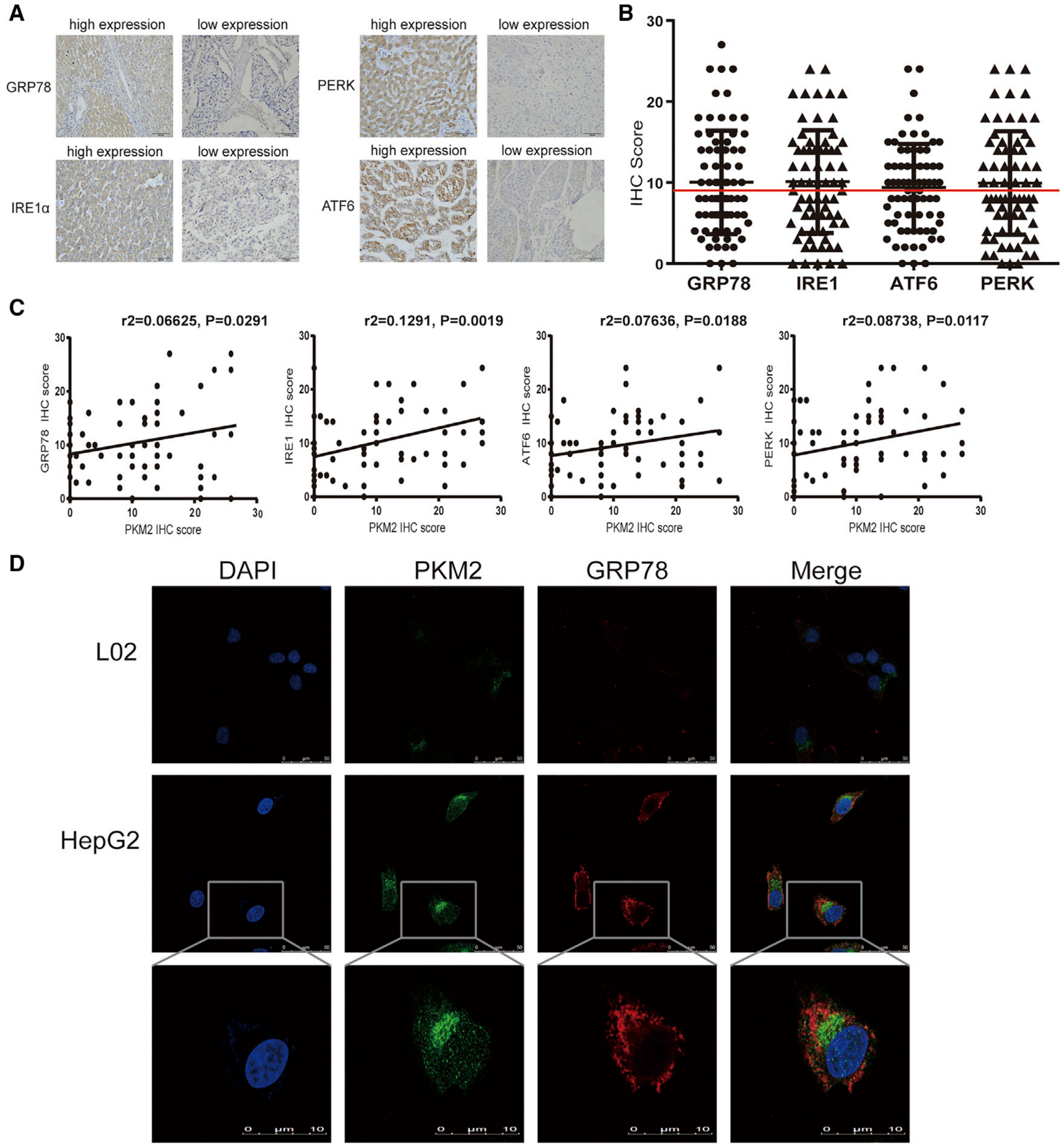

Figure 3. Overexpressed PKM2 is closely related to activated ER stress in HCC

(A and B) Expression levels of PERK, GRP78, IRE1α, and ATF6 in HCC tissue samples were examined by immunohistochemistry (IHC) analysis and semiquantitative analysis by IHC score (200× magnification). (C) Correlation analysis between PKM2 expression and ER stress-related protein expression. (D) Immunofluorescence analysis of the localization and expression of PKM2 with GRP78 in HepG2 and L02 cells (40× and 200× magnification, respectively).

sequence that match the seed sequence of miR-188-5p (Figure 6A), suggesting that hnRNPA2B1 is potential target gene of miR-188-5p. To confirm that miR-188-5p can regulate hnRNPA2B1 expression, western blot and qRT-PCR assays were performed to detect the protein and mRNA expression levels of hnRNPA2B1, respectively, in HepG2 cells after transfecting with miR-188-5p mimic and miR-188-5p-NC. In comparison with the miR-188-5p-NC groups, we found that hnRNPA2B1 was apparently decreased not only at the protein level but also at the mRNA level in the mimic group (Figures 6B–6D). Notably, the dual-luciferase assays showed that compared with 293T cells that were transfected with miR-188-5p-NC or mutated type (mut) 3′ UTR of hnRNPA2B1, the luciferase activity of 293T cells that were co-transfected with miR-188-5p mimics and wild-type (WT) 3′ UTR of hnRNPA2B1 was significantly reduced (Figure 6E). We further explored the expression of hnRNPA2B1 in 72 cases of HCC tissues and paired

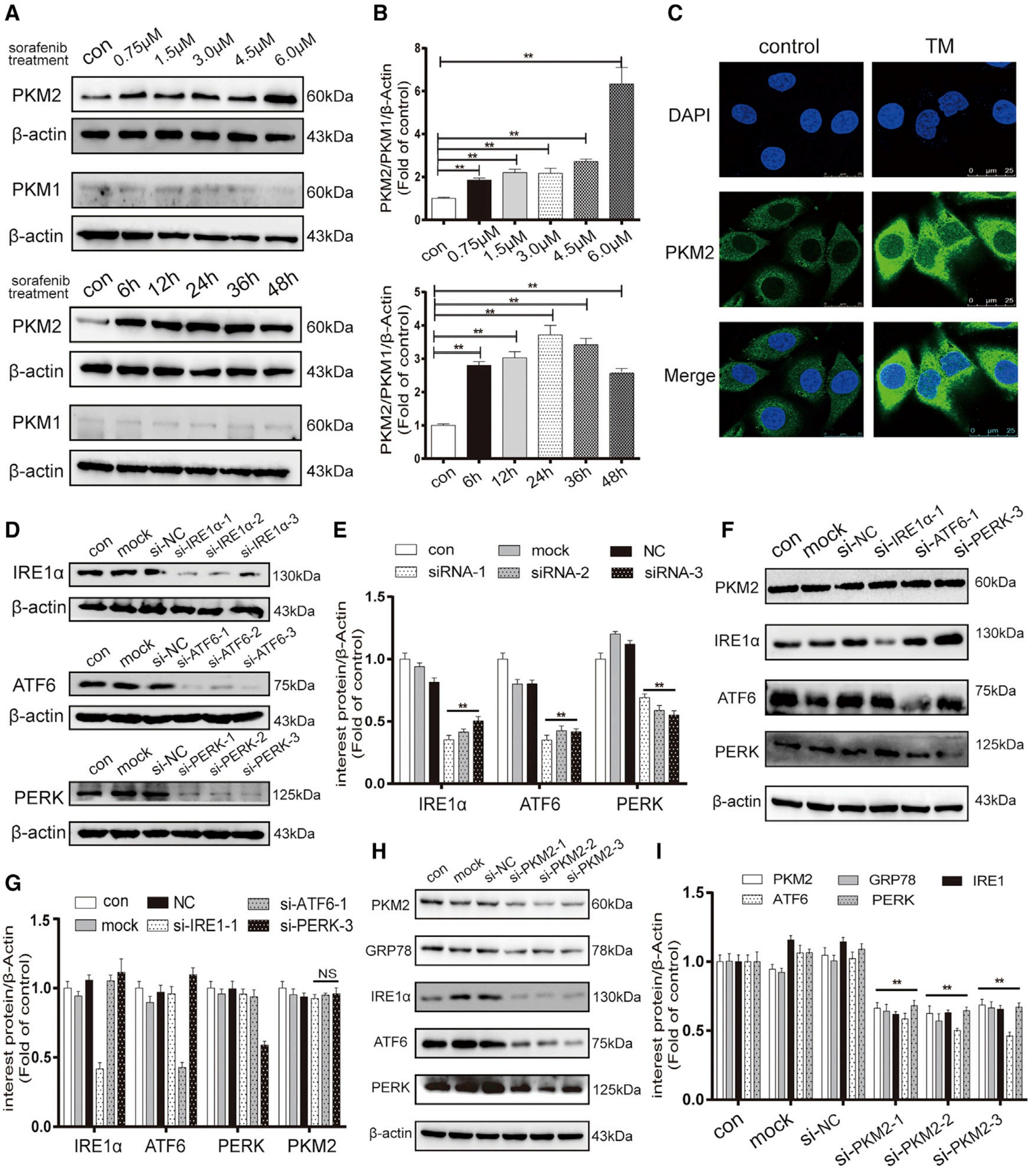

Figure 4. Activated ER stress in HCC triggers the expression of PKM2

(A and B) PKM2 and PKM1 protein levels were detected by western blot in HepG2 cells exposed to gradient concentrations of TM for 24 h and TM at 3 μM for gradient time. (C) Immunofluorescence analysis of PKM2 expression after HepG2 cells treated with TM (3 μM, 24 h) (80× magnification). (D and E) Western blot analysis of the knockdown efficiency of 3 UPR pathways. (F and G) PKM2, IRE1α, ATF6, and PERK protein expressions were determined by western blot after HepG2 cells transfected with the most effective UPR pathways siRNA for 24 h. ns, no statistical difference. (H and I) Western blot analysis of PKM2 and ER stress-related protein expression when Huh7 cells were transfected with 3 PKM2 interfering sequences for 24 h. **p < 0.01 (n = 3).

adjacent normal liver tissues by immunohistochemical analysis and found that the protein level of hnRNPA2B1 was increased in the HCC group compared with corresponding normal liver tissues and that the hnRNPA2B1 IHC score of tumor tissues was evidently higher than that in adjacent tissues (IHC scores ≥10 means hnRNPA2B1 high expression, 38/72 versus 1/72) (Figures 6F and 6G). Overall, these results manifested that hnRNPA2B1 is a direct target of miR-188-5p.

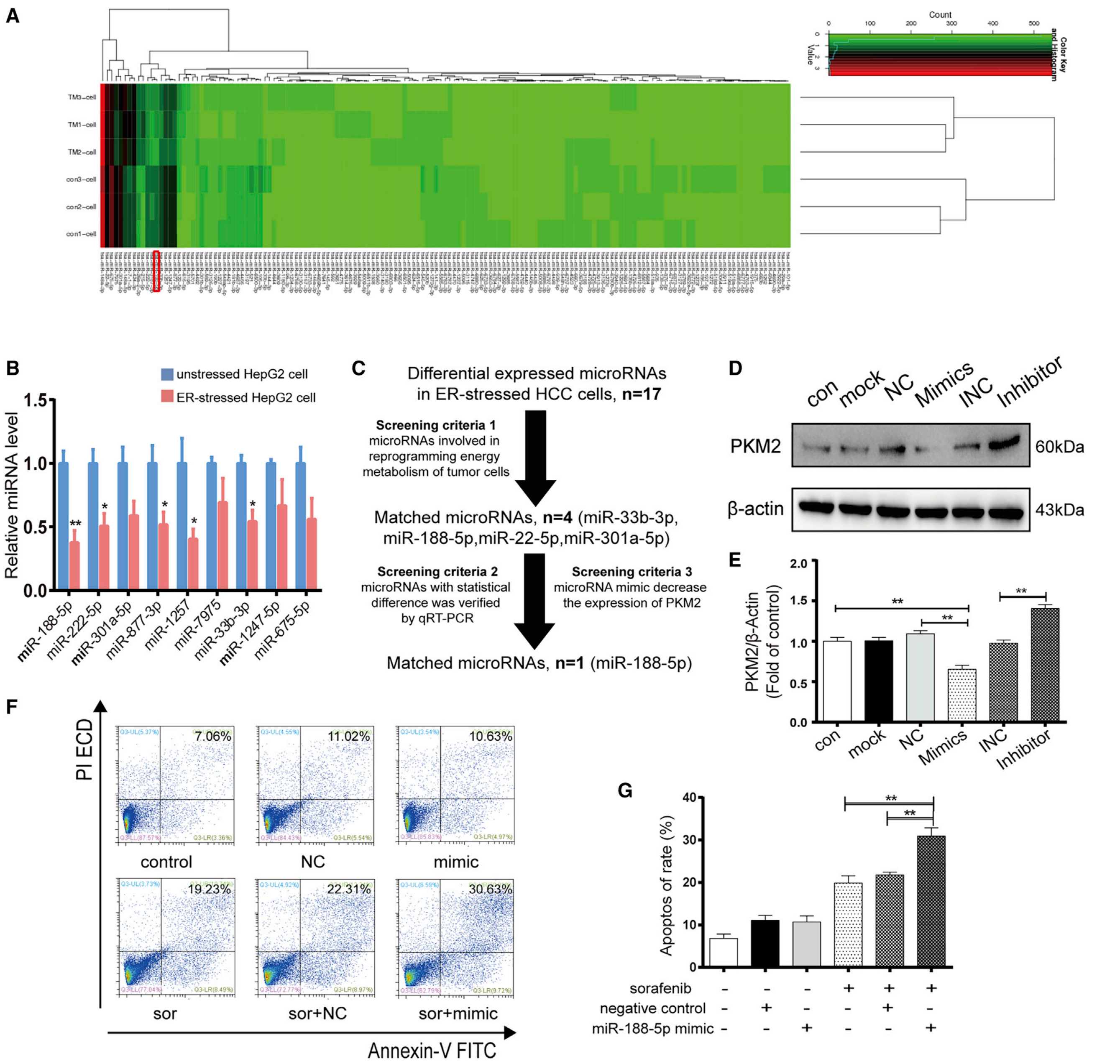

Figure 5. Activation of ER stress regulates the expression of PKM2 through miR-188-5p in HCC

(A) The heatmap of miRNAs expressed in con-HepG2 cells and TM-HepG2 cells (3 μM, 24 h). (B) qRT-PCR analysis of the levels of differentially expressed miRNAs after treating with TM (3 μM, 24 h). (C) Schematic diagrams of miRNAs found to be critical in regulating PKM2 expression. (D and E) expression levels of PKM2 were detected by western blot in HepG2 cells transfecting with miR-188-5p mimic, inhibitors, and their corresponding controls for 24 h. (F and G) Analysis of the apoptosis rate of HepG2 cells treated with sorafenib (10 μM, 24 h) after transfecting with miR-188-5p mimic or negative control for 24 h was performed by flow cytometry. *$p < 0.05$, **$p < 0.01$ ($n = 3$).

ER stress promotes sorafenib resistance via the miR-188-5p/hnRNPA2B1/PKM2 pathway in HCC

To investigate whether hnRNPA2B1 is the mediator of ER stress up-regulating PKM2 expression, first, IHC analysis of 72 HCC specimens demonstrated that most HCC patients with a high expression of hnRNPA2B1 also highly express PKM2 (27/38, $p = 0.001$) and ATF6 (27/38, $p = 0.002$), PERK (26/38, $p = 0.000$), GRP78 (23/38, $p = 0.032$), and IRE1 (31/38, $p = 0.000$) (Table S2). This finding suggests that hnRNPA2B1 is closely related to PKM2 expression and ER stress. Second, we clarify the influence of ER stress on the expression of hnRNPA2B1. Western blot results revealed that the expression of hnRNPA2B1 was upregulated with the increasing concentration of

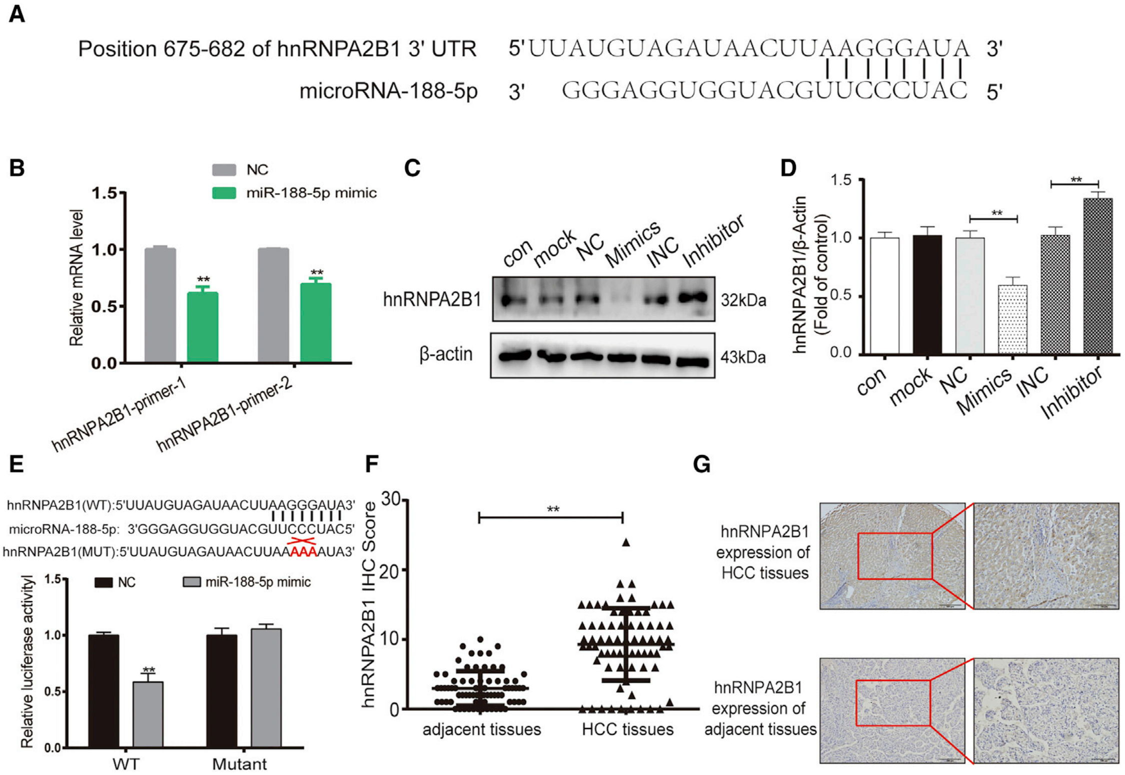

Figure 6. hnRNPA2B1 is a direct target of miR-188-5p

(A) hnRNPA2B1 mRNAs contain a complementary 3′ UTR sequence that matches the seed sequence of miR-188-5p. (B) Quantitative PCR analysis of the levels of hnRNPA2B1 mRNA after HepG2 cells transfected with miR-188-5p mimic or negative control for 24 h. (C and D) Expression levels of hnRNPA2B1 were determined by western blot in HepG2 cells transfected with miR-188-5p mimic, inhibitors, and their corresponding controls for 24 h. (E) miR-188-5p downregulates the luciferase reaction intensity of plasmids, which consist of wild-type 3′ UTR of hnRNPA2B1. (F and G) Expression of hnRNPA2B1 in HCC specimens were semiquantitatively analyzed by IHC (100× and 200× magnification). **$p < 0.01$ (n = 3).

the classic ER stress stimulator TM (Figures 7A and 7B). Third, the HNRNPs family was found to be involved in mRNA translation and splicing.[27] We must determine whether hnRNPA2B1 can mediate the alternative *PKM* mRNA splicing to regulate the expression of PKM2 and PKM1. Western blot results showed that the expression of PKM2 was evidently decreased and PKM1 was accordingly upregulated in HepG2 cells after silencing the expression of hnRNPA2B1 with siRNA (Figures 7C–7E). This result indicates that hnRNPA2B1 acts as an RNA-binding protein to mediate the alternative splicing of *PKM* mRNA into PKM2 instead of PKM1. Fourth, we investigated whether hnRNPA2B1 mediates the regulation of PKM2 by miR-188-5p. hnRNPA2B1 knockdown HpeG2 cells by a specific short hairpin RNA (shRNA) were transfected with miR-188-5p inhibitor and INC (inhibitor negative control). Immunoblotting analysis showed that hnRNPA2B1 depletion offset the effect of miR-188-5p inhibitor upregulating the expression of PKM2 (Figures 7F and 7G). Consistently, the overexpression of hnRNPA2B1 reversed the effect of miR-188-5p mimic downregulating the expression of PKM2 (Figures 7H and 7I). These data indicate that hnRNPA2B1 functions as a mediator involved in the process of miR-188-5p regulation of PKM2 expression. Finally, it has been confirmed that hnRNPA2B1 was highly expressed in liver tumor tissues. We further explored the role of hnRNPA2B1 in sorafenib resistance and the connection between its protein levels and clinical features. Clinical data statistics showed that HCC patients with a high expression of hnRNPA2B1 had poorer differentiation of HCC tissues (poor differentiation, 36.8% versus 11.8%, $p = 0.026$) and shorter overall survival (median survival, 27.5 months versus 6.0 months, $p = 0.0471$) (Figure 7J; Table S3). Meanwhile, the flow cytometry apoptosis assay results showed that downregulating the expression of hnRNPA2B1 using siRNA could enhance the apoptosis rate of HepG2 cells induced by sorafenib (Figures 7K and 7L). In conclusion, these results demonstrate that hnRNPA2B1, which is the direct target gene of miR-188-5p, mediates the process of ER stress upregulating PKM2 expression and is related to some malignant clinicopathological behaviors of HCC, including poorer differentiation of HCC tissues, shorter overall survival, and sorafenib resistance.

DISCUSSION

Studies have demonstrated that sorafenib resistance of HCC is associated with special tumor microenvironments, including hypoxia, inflammation, and oxidative stress, and malignant biological

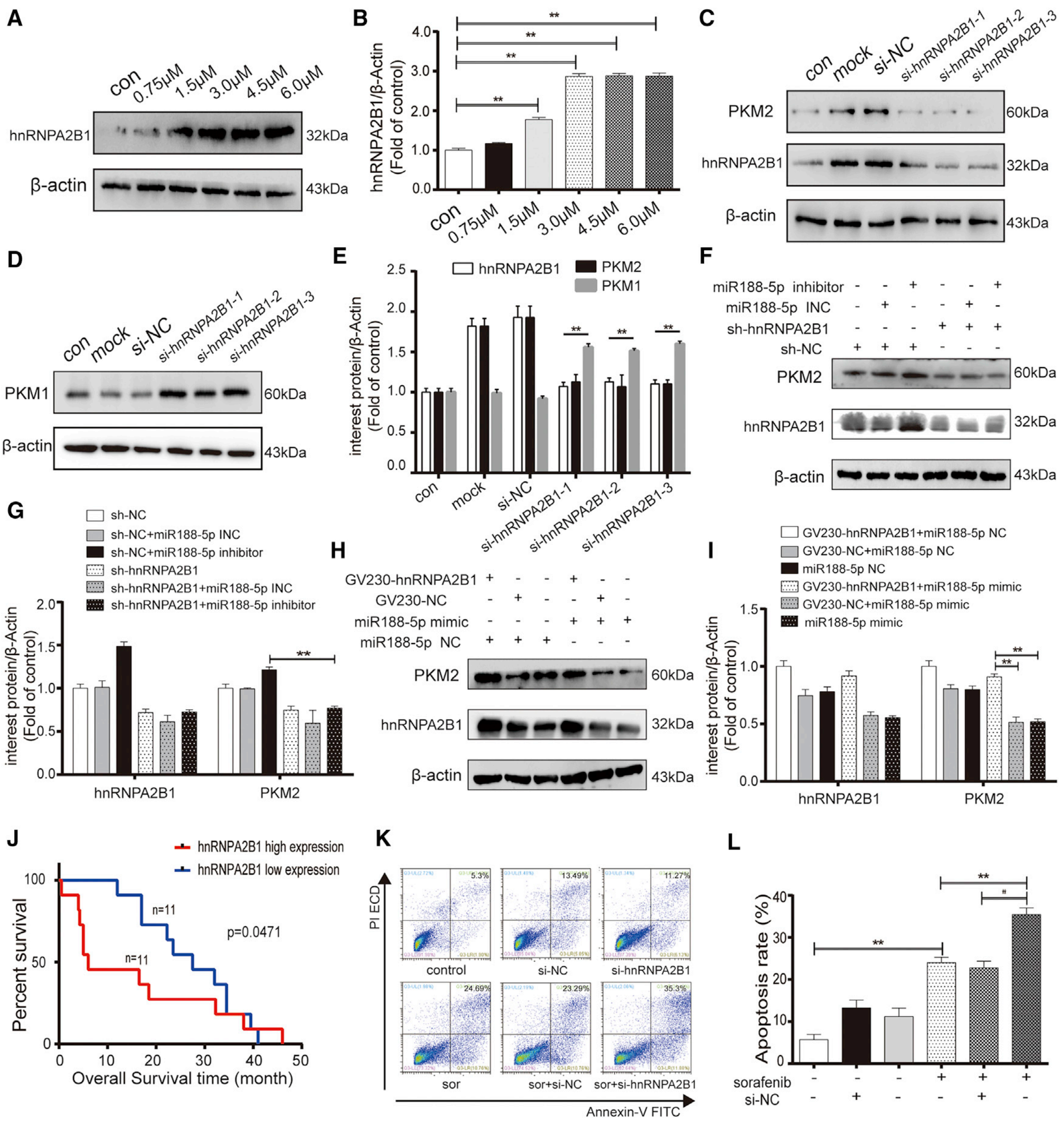

Figure 7. ER stress promotes sorafenib resistance via the miR-188-5p/hnRNPA2B1/PKM2 pathway in HCC

(A and B) The protein levels of hnRNPA2B1 were determined by western blot in HepG2 cells exposed to different concentrations of TM for 24 h. (C–E) Representative western blotting results of PKM2 and hnRNPA2B1 when HepG2 cells were transfected with 3 hnRNPA2B1 interfering sequences for 24 h. (F–I) Expression of PKM2 and hnRNPA2B1 was detected by western blot in hnRNPA2B1 knockdown HepG2 cells transfected with miR-188-5p inhibitors/INC and in hnRNPA2B1 overexpression HepG2 cells transfected with miR-188-5p mimic/NC. (J) The Kaplan-Meier method was applied in overall survival analysis between the hnRNPA2B1 high-expression and hnRNPA2B1 low-expression groups. (K and L) The apoptosis rate of HepG2 cells treated with sorafenib (10 μM, 24 h) after transfecting with si-hnRNPA2B1 or negative control for 24 h were detected by flow cytometry analysis. **$p < 0.01$ ($n = 3$).

behaviors of tumor cells involve epithelial-mesenchymal transitions, autophagy, apoptosis resistance, and energy metabolism reprogramming.[28,29] ER stress is widely regarded as a favorable factor to promote tumor cell survival. Our previous study found that ER stress was involved in sorafenib resistance of HCC to a certain extent,[8] while the precise mechanism by which ER stress leads to sorafenib resistance is not fully understood. In this study, we revealed that the therapeutic mechanism of ER stress promoting sorafenib resistance may be related to PKM2 upregulation via miR-188-5p/hnRNPA2B1.

Energy metabolism reprogramming also has been reported to promote the survival and chemotherapy resistance of tumor cells.[30]

The role of energy metabolism reprogramming, especially abnormally activated glycolysis mediated by continuous activation of PKM2 in sorafenib resistance of tumor cells, remains unclear. Studies have shown that PKM2 is overexpressed in several types of tumors, including lung cancer, breast carcinoma, and colorectal carcinoma, and is closely related to cell proliferation, apoptosis resistance, invasion, and migration.[19,20] Our research clearly demonstrated that PKM2 was significantly overexpressed in hepatocellular carcinoma; on the contrary, its isoform PKM1, which is the expression product of *PKM* gene in normal liver cells, is hardly expressed in HCC. PKM2 plays a critical role in apoptosis resistance and chemotherapy resistance. For instance, recent research has elaborated that the silence of PKM2 increases the sensitivity of bladder cancer to pirarubicin (THP) *in vivo* and *in vitro* by activating AMPK and inhibiting STAT3.[21] Furthermore, our study found that overexpressed PKM2 in HCC had a certain contribution to sorafenib resistance. These findings suggest that overexpressed and fully activated PKM2 in HCC could accelerate the process of glycolysis to provide hepatoma cells with adequate ATP in an immediate and effective manner, so that some hepatoma cells could escape the apoptosis induced by sorafenib. However, whether ER stress increases sorafenib resistance by regulating the energy metabolism reprogramming of tumor cells is unknown.

ER stress has been reported to regulate intracellular glucose and lactate metabolism by reducing the expression of GLUT1 (glucose transporter 1) on the cell surface to increase normal cell death.[31] Whether ER stress has an effect on glucose metabolism reprogramming, especially the expression of *PKM* genes, is our main research direction. We first found that PKM2 was strongly correlated with ER stress in liver cancer tissue specimens; moreover, we found that the ER stress stimulator TM increased the expression of PKM2 in liver cancer cells, while silencing PKM2 with siRNA reduced the expression of ER stress marker proteins GRP78, PERK, IRE1, and ATF6. ER stress upregulated the expression of PKM2, suggesting that ER stress as an induced factor of cellular glucose metabolism reprogramming fully activates glycolysis, allows tumor cells to obtain ATP directly and efficiently, and supplies the stressed cells with compensative energy that need to be consumed in the degradation of misfolded and unfolded polypeptide fragments. The overexpressed PKM2 positive feedback activates ER stress may make the aerobic glycolysis process continuous.

Moderate ER generally regulates the expression of related genes through the corresponding transcription factors downstream of the three UPR pathways, thereby alleviating the stress caused by intracellular and extracellular pathological factors.[32] ER stress triggers the transcription of a long-chain non-coding RNA GOLGA2P10 through the PERK/ATF4/CHOP pathway. Upregulation of GOLGA2P10 makes tumor cells avoid the cytotoxic effects of sustaining ER stress in the tumor microenvironment.[33] Whether ER stress activates PKM2 through any of the three UPR pathways has not been reported. In the process of exploring the mechanism of ER stress upregulating PKM gene expression, we observed that the activation of PKM2 depends on none of the three URP pathways.

ER stress was reported to affect the biological behavior of cells through inducing or downregulating the expression of miRNAs.[34,35] The differentially expressed miRNAs indirectly regulate the proliferation, differentiation, and apoptosis of tumor cells that is achieved by silencing the expression of related target genes. miR-27a that upregulate in colorectal cancer is closely related to glycolysis mediating chemotherapy resistance through hampering AMPK and enhancing mammalian target of rapamycin (mTOR) signaling.[36] Studies on whether miRNAs are involved in the regulation of PKM2 expression by ER stress are limited. In our present study, we observed that 17 miRNAs were differentially expressed in HCC cells under ER stress, 4 of which were involved in energy metabolism reprogramming of tumor cells—miR-188-5p, miR-33b-3p, miR-22-5P, and miR-301a-5p.[24–26] Moreover, our results showed that the downregulation of miR-188-5p was most significant in HCC cells with ER stress. Recent studies have reported that miR-188-5p acts as a tumor suppressor that is downregulated in gastric cancer, prostate cancer, and HCC, and inhibits the proliferation, invasion, and metastasis of tumor cells.[37–39] miR-188-5p has been shown to regulate the activation of rate-limiting enzymes (hexokinase2 [HK2]) in the glycolysis process to enhance the proliferation, migration, and glycolysis of HCC cells.[40] We further explored whether miR-188-5p, which downregulated in HCC cells under ER stress, reduces the expression of PKM2. Our present study revealed that miR-188-5p could downregulate the expression of PKM2 and increase HCC cell apoptosis induced by sorafenib. Collectively, our results suggested that activated ER stress in liver cancer tissue downregulated tumor suppressor miR-188-5p to relieve the expression of PKM2 to mediate sorafenib resistance.

Bioinformatics analysis has revealed that PKM2 is not the direct target gene of miR-188-5p. Whether miR-188-5p regulates the activation of PKM2 through targeting its direct target genes is our research direction. Among the predicted target genes of miR-188-5p, we found the hnRNP family, including hnRNPA0, hnRNPA1, and hnRNPA2B1, may regulate the selective expression of *PKM* genes by splicing RNA.[41] The hnRNP family was reported to play a critical role in the specific splicing of PKM mRNA, which is able to increase the ratio of PKM2:PKM1 and enhance aerobic glycolysis in colorectal cancer cells.[27] In the present study, we further identified that *hnRNPA2B1* was the direct target gene of miR-188-5p through dual-luciferase reporter and bioinformatics analysis. hnRNPA2B1 is an RNA-binding protein involved in various biological processes such as processing of heterogeneous nuclear RNA into mature mRNA, RNA splicing, transactivation of gene expression, and regulation of protein translation.[41] Several studies revealed that activated hnRNPA2B1 promotes tumor growth and malignant capability in ovarian and lung cancers.[42,43] What we need to explore further is whether hnRNPA2B1 acts as a mediator of ER stress regulating the activation of PKM2. It has

been proved that hnRNPA2B1 is involved in the regulation of GLUT1 and PKM2 mRNAs, making pancreatic cancer cells sensitive to glycolysis inhibition.[44] Simultaneously, there exists a close relationship between ER stress and the expression of the hnRNP family. ER stress in hepatic stellate cells promotes liver fibrosis via PERK-mediated degradation of hnRNPA1.[45] Our data provided evidence that hnRNPA2B1 could be induced by ER stress and be involved in the regulation of PKM2. In short, our results suggest that hnRNPA2B1 is the direct target of miR-188-5p under ER stress and also is the mediator of miR-188-5p in regulating PKM2 expression. Upregulation of hnRNPA2B1 in HCC, when induced by miR-188-5p loss under ER stress, activates PKM2 and makes HCC cells obtain survival advantage and sorafenib resistance.

In conclusion, this study demonstrated the correlation between activated ER stress and the overexpressed PKM2 in HCC, both of which are involved in sorafenib resistance, and revealed that ER stress could upregulate the expression of PKM2 through miR-188-5p/hnRNPA2B1. The downregulation of tumor suppressor miR-188-5p in ER-stressed HCC cells could increase PKM2-mediated sorafenib resistance by directly targeting hnRNPA2B1. Therefore, targeting the miR-188-5p/hnRNPA2B1/PKM2 pathway and ER stress may prove instrumental in overcoming sorafenib resistance in HCC therapy.

MATERIALS AND METHODS

Reagents and drugs

The following reagents were used in this research: drugs including TM (#T7765) and sorafenib (#SRP0702) (Sigma-Aldrich, USA); primary antibodies against PERK (#2156), hnRNPA2B1 (#6196), ATF6 (#1034R) (Bioworld Technology, USA), IRE1α (#14C10), PKM1 (#D30G6) (Cell Signaling Technology, USA), GRP78 (#54027) (Arigo Biolaboratories, China), and PKM2 (#60268-1-lg) (Proteintech, China).

Cell culture

Hepatocyte cell line L02 and human liver cancer cell lines SMMC-7721, HepG2, Hep3B, and Huh7 were obtained from the Chinese Academy of Sciences. These cells were cultured in a basic nutrient solution consisting of high-sugar DMEM, fetal bovine serum (FBS) (10%), and dual antibiotics (penicillin and streptomycin, 1%), and were placed in an incubator at 37°C and 5% CO_2.

Human HCC specimens

Human primary HCC tissue specimens were obtained from patients who experienced liver cancer radical or palliative excision at the First Affiliated Hospital of Anhui Medical University from 2012 to 2017. No other interventions against HCC tissue before surgery were performed. Clinical and pathological information, including gender, age, cirrhotic condition, hepatitis infection, tumor volume, degree of differentiation of tumor cell, clinical stage, and overall survival, were collected through medical records and medical history consulting and telephone follow-up. The informed consent of these patients has been guaranteed. The study protocol was carried out in accordance with the Declaration of Helsinki and approved by the ethics committee of Anhui Medical University.

Construction of HCC tissue chip

The excised liver cancer tissues and adjacent tissues after formalin fixation and paraffin embedding were selected to determine the target region based on H&E staining. Two typical tissues (1 mm) were cut from the target regions of each specimen and then embedded in the recipient paraffin block for secondary embedding to make tissue chips.

Immunohistochemical analysis

After dewaxing, hydrating, and permeating, the tissue chip was blocked with inherent peroxidase activity by reacting with 3% hydrogen peroxide. Then, the chip was subjected to antigen repair to expose the epitope and placed in 5% serum to block non-specific proteins. The tissue microarray was successively incubated with specific primary antibody and corresponding secondary antibody, followed by moderate 3,3′-diaminobenzidine (DAB) dye solution for color rendering and hematoxylin for counterstaining. Finally, dehydration was carried out in a gradient concentration alcohol. Staining intensity and the number of positively stained cells are two critical indicators of immunohistochemical score. The staining intensity is divided into 0–3 points and the cell positive rate is divided into 0–9 points (e.g., 0 represents 0%–10% positive rate, 1 represents 10%–20% positive rate). We identify that the final IHC score of cancer tissue or adjacent tissue is the product of the scores of these two indicators.[46] Furthermore, we identified the IHC score ≥ 10 as high expression and the IHC score <10 as low expression based on median expression.

Western blot analysis

Cells were lysed in lysis buffer with phosphatase inhibitors and protease inhibitors for 30 min on ice. Proteins were separated by electrophoresis and transferred to polyvinylidene fluoride (PVDF) membranes. Protein-loaded membranes were incubated with primary antibodies overnight at 4°C. Goat anti-rabbit secondary antibody was incubated for 2 h at 37°C. Protein expressions were reflected by the grayscale value of each protein band, which was quantitatively acquired and analyzed by ImageJ software (National Institutes of Health, USA).

MTT assay

The adherent cells were digested into cell suspensions, which are planted in a 96-well plate at 1,000–10,000 cells/well, with a volume of 200 μL/well. After treatment on the basis of the experimental purpose and requirements, the cells were added with 20 μL MTT and continued culturing for 4 h in the incubator. We terminated the culture, carefully discarded the supernatant from the wells, added 150 μL DMSO to each well, and shook for 10 min to fully dissolve the sediments. The absorbance of the solution of every well was obtained from an enzyme-linked immunoassay monitor with a wavelength of 490 nm (Bio-Tek Instruments, USA).

Flow cytometry

The single cell suspension was processed from the adherent cells and resuspended with 400 μL binding buffer. Two fluorescent dyes (annexin V-FITC and PI, 5 μL, BD Biosciences, USA) were added to each tube successively and incubated for 15 min. The fluorescence intensity of FITC and PI were detected. All of the data were collected using flow cytometry (Beckman Coulter, USA) and analyzed with CytoExpert.

Colony formation assay

HepG2 and Huh7 cells were treated with sorafenib after transfecting PKM2 siRNA or overexpression plasmid. These treated cells were collected and planted on the 6-well plates at a density of 1×10^3 cells/well for 14 days to allow colony formation; 4% polyformaldehyde and 1% Crystal Violet were used for fixation and staining, respectively.

Immunofluorescence

The suspending cells were placed on slides in a sterile dish for adherent growth. Cell-adhered coverslips were permeabilized in 0.5% Triton X-100 for 10 min and blocked in 5% BSA for 90 min. Subsequently, the cells on the slide were fully immunoreactive with the objective primary antibody (dilution 1:100) overnight at 4°C, followed by incubating with a corresponding secondary antibody (dilution 1:100). Subsequently, the cells were reacted with DAPI in the dark for 5 min and visualized under a laser confocal microscope.

Quantitative real-time-PCR

Total RNA was isolated from HepG2 cells by TRIzol reagent (Invitrogen, USA), and we detected the concentration of RNA by Nanodrop 2000 (Thermo Scientific, USA). Quantitative real-time-PCR analyses for the mRNAs of hnRNPA2B1 and β-actin and the miRNAs of miR33b-3p, miR-188-5p, miR222-5p, miR301a-5p, miR675-5p, miR877-3p, miR1247-5p, miR1257, and miR7975 were disposed under a standard protocol. The sequences of all of the primers are listed in Table S4.

siRNA and plasmid transfection

Liver cancer cells were planted in sterile dishes for adherent growth and then transfected with GV230-PKM2 or GV230-Control and siRNA (Gene Pharmaceutical Technology, China) using LipofectamineTM2000 (Invitrogen, USA) in serum-free medium (GIBCO, USA) according to the operational guidelines and replacing the culture medium after 6 h. The efficiency of overexpressing and interfering was identified by western blot analysis after 24 h.

Luciferase assay

The hnRNPA2B1 3′ UTR sequences containing the presumed miR-188 binding sites or mutated binding sites were inserted into the p-MIR-reporter plasmid (Ambion). Then, 293T cells were co-transfected with a β-galactosidase (β-gal) expression plasmid (Ambion, USA). A firefly luciferase reporter plasmid, miR-188, mimics negative control. The β-gal plasmid was used as a transfection control. Luciferase activity was measured 24 h after transfection using a luciferase assay kit (Promega, USA).

Detection of miRNAs by high-throughput sequencing technology

Total RNA was extracted from 3 groups of untreated HepG2 cells and 3 groups of HepG2 cells treated with 3 μM TM for 24 h with Trizol reagent, $\sim 2 \times 10^5$ cells per group. cDNA libraries were constructed before sequencing the miRNA profiles using the Hiseq2000 platform. miRNAs with expressions changed by >2-fold, as determined by high-throughput sequencing, were used as candidate genes.

Statistical analysis

The statistical significance of differences is calculated by the Student's t test in SPSS 16.0 software (IBM SPSS Statistics, USA). Correlation between protein expression and between protein expression and clinical parameters were conducted by the χ^2 test and correlation analysis. The difference in overall survival between different groups was studied using the Kaplan-Meier analysis. $p < 0.05$ represents the statistically significant difference. Every experiment was performed three times.

SUPPLEMENTAL INFORMATION

Supplemental information can be found online at https://doi.org/10.1016/j.omtn.2021.09.014.

ACKNOWLEDGMENTS

This study was supported by the National Natural Science Foundation of China (81872047 and 82072687) and the National Natural Science Foundation of AnHui (1808085MH239). We acknowledge the members of our lab for helpful discussion and technical assistance.

AUTHOR CONTRIBUTIONS

G.S. and J.L. designed the study; B.Z., A.W., and D.L. performed the experiments; B.Z., J.C., and L.Z. analyzed the data; B.Z., J.L., and A.W. wrote the paper; and L.F. and W.W. participated in the design of the study.

DECLARATION OF INTERESTS

The authors declare no competing interests.

REFERENCES

1. Teng, Y.X., Li, M.J., Xiang, B.D., and Zhong, J.H. (2020). Tenofovir may be superior to entecavir for preventing hepatocellular carcinoma and mortality in individuals chronically infected with HBV: a meta-analysis. Gut *69*, 1900–1902.
2. Raoul, J.L., and Edeline, J. (2020). Systemic treatment of hepatocellular carcinoma: standard of care in China and elsewhere. Lancet Oncol. *21*, 479–481.
3. Reiss, K.A., Yu, S., Mamtani, R., Mehta, R., D'Addeo, K., Wileyto, E.P., Taddei, T.H., and Kaplan, D.E. (2017). Starting Dose of Sorafenib for the Treatment of Hepatocellular Carcinoma: A Retrospective, Multi-Institutional Study. J. Clin. Oncol. *35*, 3575–3581.
4. Niu, L., Liu, L., Yang, S., Ren, J., Lai, P.B.S., and Chen, G.G. (2017). New insights into sorafenib resistance in hepatocellular carcinoma: responsible mechanisms and promising strategies. Biochim. Biophys. Acta Rev. Cancer *1868*, 564–570.

5. Cubillos-Ruiz, J.R., Bettigole, S.E., and Glimcher, L.H. (2017). Tumorigenic and Immunosuppressive Effects of Endoplasmic Reticulum Stress in Cancer. Cell *168*, 692–706.

6. Feng, D., Wang, B., Wang, L., Abraham, N., Tao, K., Huang, L., Shi, W., Dong, Y., and Qu, Y. (2017). Pre-ischemia melatonin treatment alleviated acute neuronal injury after ischemic stroke by inhibiting endoplasmic reticulum stress-dependent autophagy via PERK and IRE1 signalings. J. Pineal Res. *62*, e12395.

7. Tao, S.C., Yuan, T., Rui, B.Y., Zhu, Z.Z., Guo, S.C., and Zhang, C.Q. (2017). Exosomes derived from human platelet-rich plasma prevent apoptosis induced by glucocorticoid-associated endoplasmic reticulum stress in rat osteonecrosis of the femoral head via the Akt/Bad/Bcl-2 signal pathway. Theranostics *7*, 733–750.

8. Zhou, B., Lu, Q., Liu, J., Fan, L., Wang, Y., Wei, W., Wang, H., and Sun, G. (2019). Melatonin Increases the Sensitivity of Hepatocellular Carcinoma to Sorafenib through the PERK-ATF4-Beclin1 Pathway. Int. J. Biol. Sci. *15*, 1905–1920.

9. Thorne, J.L., and Campbell, M.J. (2015). Nuclear receptors and the Warburg effect in cancer. Int. J. Cancer *137*, 1519–1527.

10. Dayton, T.L., Gocheva, V., Miller, K.M., Israelsen, W.J., Bhutkar, A., Clish, C.B., Davidson, S.M., Luengo, A., Bronson, R.T., Jacks, T., and Vander Heiden, M.G. (2016). Germline loss of PKM2 promotes metabolic distress and hepatocellular carcinoma. Genes Dev. *30*, 1020–1033.

11. Méndez-Lucas, A., Li, X., Hu, J., Che, L., Song, X., Jia, J., Wang, J., Xie, C., Driscoll, P.C., Tschaharganeh, D.F., et al. (2017). Glucose Catabolism in Liver Tumors Induced by c-MYC Can Be Sustained by Various PKM1/PKM2 Ratios and Pyruvate Kinase Activities. Cancer Res. *77*, 4355–4364.

12. Zhang, M., Zhang, H., Hong, H., and Zhang, Z. (2019). MiR-374b re-sensitizes hepatocellular carcinoma cells to sorafenib therapy by antagonizing PKM2-mediated glycolysis pathway. Am. J. Cancer Res. *9*, 765–778.

13. Kim, C., Song, H.S., Park, H., and Kim, B. (2018). Activation of ER Stress-Dependent miR-216b Has a Critical Role in *Salviamiltiorrhiza* Ethanol-Extract-Induced Apoptosis in U266 and U937 Cells. Int. J. Mol. Sci. *19*, 1240.

14. Liu, J., Fan, L., Yu, H., Zhang, J., He, Y., Feng, D., Wang, F., Li, X., Liu, Q., Li, Y., et al. (2019). Endoplasmic Reticulum Stress Causes Liver Cancer Cells to Release Exosomal miR-23a-3p and Up-regulate Programmed Death Ligand 1 Expression in Macrophages. Hepatology *70*, 241–258.

15. Jiang, X., Wang, J., Deng, X., Xiong, F., Zhang, S., Gong, Z., Li, X., Cao, K., Deng, H., He, Y., et al. (2020). The role of microenvironment in tumor angiogenesis. J. Exp. Clin. Cancer Res. *39*, 204.

16. Zhang, X., Schulz, R., Edmunds, S., Krüger, E., Markert, E., Gaedcke, J., Cormet-Boyaka, E., Ghadimi, M., Beissbarth, T., Levine, A.J., et al. (2015). MicroRNA-101 Suppresses Tumor Cell Proliferation by Acting as an Endogenous Proteasome Inhibitor via Targeting the Proteasome Assembly Factor POMP. Mol. Cell *59*, 243–257.

17. Ma, F., Zhang, L., Ma, L., Zhang, Y., Zhang, J., and Guo, B. (2017). MiR-361-5p inhibits glycolytic metabolism, proliferation and invasion of breast cancer by targeting FGFR1 and MMP-1. J. Exp. Clin. Cancer Res. *36*, 158.

18. Wang, T., Chen, G., Ma, X., Yang, Y., Chen, Y., Peng, Y., Bai, Z., Zhang, Z., Pei, H., and Guo, W. (2019). MiR-30a regulates cancer cell response to chemotherapy through SNAI1/IRS1/AKT pathway. Cell Death Dis. *10*, 153.

19. Chaneton, B., and Gottlieb, E. (2012). Rocking cell metabolism: revised functions of the key glycolytic regulator PKM2 in cancer. Trends Biochem. Sci. *37*, 309–316.

20. Yang, W., Xia, Y., Cao, Y., Zheng, Y., Bu, W., Zhang, L., You, M.J., Koh, M.Y., Cote, G., Aldape, K., et al. (2018). EGFR-Induced and PKCε Monoubiquitylation-Dependent NF-κB Activation Upregulates PKM2 Expression and Promotes Tumorigenesis. Mol. Cell *69*, 347.

21. Su, Q., Tao, T., Tang, L., Deng, J., Darko, K.O., Zhou, S., Peng, M., He, S., Zeng, Q., Chen, A.F., and Yang, X. (2018). Down-regulation of PKM2 enhances anticancer efficiency of THP on bladder cancer. J. Cell. Mol. Med. *22*, 2774–2790.

22. Walter, F., Schmid, J., Düssmann, H., Concannon, C.G., and Prehn, J.H. (2015). Imaging of single cell responses to ER stress indicates that the relative dynamics of IRE1/XBP1 and PERK/ATF4 signalling rather than a switch between signalling branches determine cell survival. Cell Death Differ. *22*, 1502–1516.

23. Guo, J., Yang, Z., Yang, X., Li, T., Liu, M., and Tang, H. (2018). miR-346 functions as a pro-survival factor under ER stress by activating mitophagy. Cancer Lett. *413*, 69–81.

24. Alamoudi, A.A., Alnoury, A., and Gad, H. (2018). miRNA in tumour metabolism and why could it be the preferred pathway for energy reprograming. Brief. Funct. Genomics *17*, 157–169.

25. Zhang, L.F., Jiang, S., and Liu, M.F. (2017). MicroRNA regulation and analytical methods in cancer cell metabolism. Cell. Mol. Life Sci. *74*, 2929–2941.

26. Chen, B., Li, H., Zeng, X., Yang, P., Liu, X., Zhao, X., and Liang, S. (2012). Roles of microRNA on cancer cell metabolism. J. Transl. Med. *10*, 228.

27. Lan, Z., Yao, X., Sun, K., Li, A., Liu, S., and Wang, X. (2020). The Interaction Between lncRNA SNHG6 and hnRNPA1 Contributes to the Growth of Colorectal Cancer by Enhancing Aerobic Glycolysis Through the Regulation of Alternative Splicing of PKM. Front. Oncol. *10*, 363.

28. Chen, J., Jin, R., Zhao, J., Liu, J., Ying, H., Yan, H., Zhou, S., Liang, Y., Huang, D., Liang, X., et al. (2015). Potential molecular, cellular and microenvironmental mechanism of sorafenib resistance in hepatocellular carcinoma. Cancer Lett. *367*, 1–11.

29. Xia, H., Ooi, L.L., and Hui, K.M. (2013). MicroRNA-216a/217-induced epithelial-mesenchymal transition targets PTEN and SMAD7 to promote drug resistance and recurrence of liver cancer. Hepatology *58*, 629–641.

30. Shanmugasundaram, K., Nayak, B.K., Friedrichs, W.E., Kaushik, D., Rodriguez, R., and Block, K. (2017). NOX4 functions as a mitochondrial energetic sensor coupling cancer metabolic reprogramming to drug resistance. Nat. Commun. *8*, 997.

31. Wang, X., Eno, C.O., Altman, B.J., Zhu, Y., Zhao, G., Olberding, K.E., Rathmell, J.C., and Li, C. (2011). ER stress modulates cellular metabolism. Biochem. J. *435*, 285–296.

32. Maillo, C., Martín, J., Sebastián, D., Hernández-Alvarez, M., García-Rocha, M., Reina, O., Zorzano, A., Fernandez, M., and Méndez, R. (2017). Circadian- and UPR-dependent control of CPEB4 mediates a translational response to counteract hepatic steatosis under ER stress. Nat. Cell Biol. *19*, 94–105.

33. Wu, M.Z., Fu, T., Chen, J.X., Lin, Y.Y., Yang, J.E., and Zhuang, S.M. (2020). LncRNA GOLGA2P10 is induced by PERK/ATF4/CHOP signaling and protects tumor cells from ER stress-induced apoptosis by regulating Bcl-2 family members. Cell Death Dis. *11*, 276.

34. Zhao, N., Mi, L., Zhang, X., Xu, M., Yu, H., Liu, Z., Liu, X., Guan, G., Gao, W., and Wang, J. (2018). Enhanced MiR-711 transcription by PPARγ induces endoplasmic reticulum stress-mediated apoptosis targeting calnexin in rat cardiomyocytes after myocardial infarction. J. Mol. Cell. Cardiol. *118*, 36–45.

35. Heindryckx, F., Binet, F., Ponticos, M., Rombouts, K., Lau, J., Kreuger, J., and Gerwins, P. (2016). Endoplasmic reticulum stress enhances fibrosis through IRE1α-mediated degradation of miR-150 and XBP-1 splicing. EMBO Mol. Med. *8*, 729–744.

36. Barisciano, G., Colangelo, T., Rosato, V., Muccillo, L., Taddei, M.L., Ippolito, L., Chiarugi, P., Galgani, M., Bruzzaniti, S., Matarese, G., et al. (2020). miR-27a is a master regulator of metabolic reprogramming and chemoresistance in colorectal cancer. Br. J. Cancer *122*, 1354–1366.

37. Peng, Y., Shen, X., Jiang, H., Chen, Z., Wu, J., Zhu, Y., Zhou, Y., and Li, J. (2018). miR-188-5p Suppresses Gastric Cancer Cell Proliferation and Invasion via Targeting ZFP91. Oncol. Res. *27*, 65–71.

38. Zhang, H., Qi, S., Zhang, T., Wang, A., Liu, R., Guo, J., Wang, Y., and Xu, Y. (2015). miR-188-5p inhibits tumour growth and metastasis in prostate cancer by repressing LAPTM4B expression. Oncotarget *6*, 6092–6104.

39. Ma, J., Qin, C., Yuan, Z., and Liu, S. (2019). LncRNA PAPAS promotes hepatocellular carcinoma by interacting with miR-188-5p. J. Cell. Biochem. *120*, 13494–13500.

40. Ding, Z., Guo, L., Deng, Z., and Li, P. (2020). Circ-PRMT5 enhances the proliferation, migration and glycolysis of hepatoma cells by targeting miR-188-5p/HK2 axis. Ann. Hepatol. *19*, 269–279.

41. Alarcón, C.R., Goodarzi, H., Lee, H., Liu, X., Tavazoie, S., and Tavazoie, S.F. (2015). HNRNPA2B1 Is a Mediator of m(6)A-Dependent Nuclear RNA Processing Events. Cell *162*, 1299–1308.

42. Xuan, Y., Wang, J., Ban, L., Lu, J.J., Yi, C., Li, Z., Yu, W., Li, M., Xu, T., Yang, W., et al. (2016). hnRNPA2/B1 activates cyclooxygenase-2 and promotes tumor growth in human lung cancers. Mol. Oncol. *10*, 610–624.

43. Yang, Y., Wei, Q., Tang, Y., Yuanyuan, W., Luo, Q., Zhao, H., He, M., Wang, H., Zeng, Q., Lu, W., et al. (2020). Loss of hnRNPA2B1 inhibits malignant capability and promotes apoptosis via down-regulating Lin28B expression in ovarian cancer. Cancer Lett. *475*, 43–52.

44. Brandi, J., Cecconi, D., Cordani, M., Torrens-Mas, M., Pacchiana, R., Dalla Pozza, E., Butera, G., Manfredi, M., Marengo, E., Oliver, J., et al. (2016). The antioxidant uncoupling protein 2 stimulates hnRNPA2/B1, GLUT1 and PKM2 expression and sensitizes pancreas cancer cells to glycolysis inhibition. Free Radic. Biol. Med. *101*, 305–316.

45. Koo, J.H., Lee, H.J., Kim, W., and Kim, S.G. (2016). Endoplasmic Reticulum Stress in Hepatic Stellate Cells Promotes Liver Fibrosis via PERK-Mediated Degradation of HNRNPA1 and Up-regulation of SMAD2. Gastroenterology *150*, 181–193.e8.

46. Seethala, R.R., Gooding, W.E., Handler, P.N., Collins, B., Zhang, Q., Siegfried, J.M., and Grandis, J.R. (2008). Immunohistochemical analysis of phosphotyrosine signal transducer and activator of transcription 3 and epidermal growth factor receptor autocrine signaling pathways in head and neck cancers and metastatic lymph nodes. Clin. Cancer Res. *14*, 1303–1309.

OMTN, Volume *26*

Supplemental information

Endoplasmic reticulum stress promotes sorafenib resistance via miR-188-5p/hnRNPA2B1-mediated upregulation of PKM2 in hepatocellular carcinoma

Bei Zhou, Donghui Lu, Anqi Wang, Jie Cui, Li Zhang, Jian Li, Lulu Fan, Wei Wei, Jiatao Liu, and Guoping Sun

Supplemental table 1. Correlations between PKM2 and ER-stress related proteins expression in HCC patients

ER-stress related proteins expression	PKM2 expression		R^2	*P*-value
	Low (N=34)	High (N=38)		
GRP78 expression			0.0663	0.0291*
Low(N=37)	22	15		
High(N=35)	12	23		
PERK expression			0.0874	0.0117*
Low(N=37)	21	16		
High(N=35)	13	22		
IRE1α expression			0.1291	0.0019**
Low(N=32)	21	11		
High(N=40)	13	27		
ATF6 expression			0.0764	0.0188*
Low(N=33)	21	12		
High(N=39)	13	26		

GRP78: glucose-regulated protein; PERK: PKR-like ER stress kinase; IRE1α: inositol-requiring transmembrane kinase and endonuclease 1α; ATF6: activating transcription factor 6; **P < 0.01, *P < 0.05.

Supplemental table 2. Correlations between ER-stress related proteins or PKM2 and hnRNPA2B1 expression in HCC patients

ER-stress related proteins or PKM2 expression	hnRNPA2B1 expression		$\chi 2$	*P*-value
	Low (N=34)	High (N=38)		
GRP78 expression			4.573	0.032*
Low(N=37)	22	15		
High(N=35)	12	23		
PERK expression			12.642	0.000**
Low(N=37)	25	12		
High(N=35)	9	26		
IRE1 expression			22.071	0.000**
Low(N=32)	25	7		
High(N=40)	9	31		
ATF6 expression			9.242	0.002**
Low(N=33)	22	11		
High(N=39)	12	27		
PKM2 expression			10.783	0.001**
Low(N=34)	23	11		
High(N=38)	11	27		

hnRNPA2B1: heterogeneous nuclear ribonucleoprotein A2/B1. **P < 0.01, *P < 0.05.

Supplemental table 3. Clinicopathologic features of HCC patients depending on hnRNPA2B1 expression

Clinical pathological factors	Total (N=72)	hnRNPA2B1 expression		$\chi 2$	*P*-value
		Low (N=34)	High (N=38)		
Gender，N(%)				0.086	0.770
M	20(27.7)	10(29.4)	10(26.3)		
F	52(72.2)	24(70.6)	28(73.7)		
Age，N(%)				0.350	0.554
≤60 y	44(62.5)	22(64.7)	22(57.9)		
＞60 y	28(37.5)	12(35.3)	16(42.1)		
History of hepatitis, N(%)				0.009	0.992
N	25(34.7)	12(35.3)	13(34.2)		
Y	47(65.3)	22(64.7)	25(65.8)		
History of cirrhosis，				1.006	0.316
N	40(55.6)	21(61.8)	19(50.0)		
Y	32(44.4)	13(38.2)	19(50.0)		
Tumor size，N(%)				6.019	0.067
＜5cm	18(25.0)	13(38.2)	5(13.2)		
5-10cm	36(50.0)	14(41.2)	22(57.9)		
≥10cm	18(25.0)	7(20.6)	11(28.9)		
Clinical stages，N(%)				0.991	0.320
Ⅰ/Ⅱ	51(70.8)	26(76.5)	25(65.8)		
Ⅲ/Ⅳ	21(29.2)	8(23.5)	13(34.2)		
Degree of differentiation，N(%)				7.155	0.026*
high	20(27.8)	13(38.2)	7(18.4)		
moderate	34(47.2)	17(50.0)	17(44.7)		
poor	18(25.0)	4(11.8)	14(36.8)		
AFP value, N(%)				1.935	0.164
＜400 ng/mL	47(65.3)	25(73.5)	22(57.9)		
≥400 ng/mL	25(34.7)	9(26.5)	16(42.1)		

Supplemental table 4. Oligonucleotide Sequences

**Oligonucleotide**	**Sequences**	
	**Sense (5'-3')**	**Antisense (5'-3')**
mimic negative control	UUCUCCGAACGUGUCACGUTT	ACGUGACACGUUCGGAGAATT
miR-188-5p mimics	CAUCCCUUGCAUGGUGGAGGG	UUGUAGGGAACGUACCACCUC
inhibitor negative control	CAGUACUUUUGUGUAGUACAA	

miR-188-5p inhibitor	CCCUCCACCAUGCAAGGGAUG	
hnRNPA2B-primer-1	CAGGGTAGTTGAGCCAAAACG	TTCCAGACTGCCTATCGGTAA
hnRNPA2B-primer-2	AAGAAATGCAGGAAGTCCAAAGT	CTCCTCCATAACCAGGGCTAC
hsa-miR-188-5p	GCGCATCCCTTGCATGGT	AGTGCAGGGTCCGAGGTATT
miR-33b-3p	GCAGTGCCTCGGCAGTG	AGTGCAGGGTCCGAGGTATT
miR-222-5p	CGCGCTCAGTAGCCAGTGTA	AGTGCAGGGTCCGAGGTATT
hsa-miR-301a-5p	CGCGGCTCTGACTTTATTGC	AGTGCAGGGTCCGAGGTATT
hsa-miR-675-5p	GGTGCGGAGAGGGCCC	AGTGCAGGGTCCGAGGTATT
hsa-miR-877-3p	CGCGTCCTCTTCTCCCTCC	AGTGCAGGGTCCGAGGTATT
hsa-miR-1247-5p	GACCCGTCCCGTTCGTC	AGTGCAGGGTCCGAGGTATT
hsa-miR-1257	CGCGAGTGAATGATGGGTT	AGTGCAGGGTCCGAGGTATT
hsa-miR-7975	CGCGCGATCCTAGTCACG	AGTGCAGGGTCCGAGGTATT
